# WHO, HOW, AND WHEN TO PREPARE FOR CARE? PREPAREDNESS AMONG CAREGIVERS OF PERSONS LIVING WITH DEMENTIA

**DOI:** 10.1093/geroni/igad104.1369

**Published:** 2023-12-21

**Authors:** Emily Mroz, Nicole Crawford, Joan Monin

## Abstract

Our health systems rely on family caregivers to provide critical support for over six million persons living with dementia (PLWD) in the US. Caregivers, however, often report low preparedness for care roles, promoting negative outcomes for themselves and their care recipients. As the number of PLWD grows, preparing close others to perform and thrive in caregiving roles is a public health priority. This symposium provides insights on who, how, and when to prepare for the care of a PLWD. First, Falzarano et al. will discuss the individual, psychosocial, and contextual factors influencing caregivers’ use of supportive services, and implications for self-efficacy and preparedness. Mroz et. al. will introduce the concept of ‘experienced’ caregivers of PLWD, those who have been caregivers previously, and describe ways to address persisting psychological tolls and low confidence from prior care roles to prepare these caregivers for current care roles. Fox et al. will describe interaction between preparedness and family dynamics, showcasing differences in these relations across dementia- and non-dementia care roles. Vu et al. will describe how caregiver engagement in a health priorities identification process can better prepare caregivers to make values-based medical decisions on behalf of the PLWD. Crawford et al. will describe the influence of burden on caregivers’ emergency preparedness. Takeaways from these papers and Joan Monin’s discussion collectively respond to priorities outlined by the NIA IMPACT Collaboratory’s Lived Experience Panel, offering direction on best practices and needed initiatives to bolster preparedness in this caregiver population.

